# Instant *in-situ* Tissue Repair by Biodegradable PLA/Gelatin Nanofibrous Membrane Using a 3D Printed Handheld Electrospinning Device

**DOI:** 10.3389/fbioe.2021.684105

**Published:** 2021-07-28

**Authors:** Hongrang Chen, Haitao Zhang, Yun Shen, Xingliang Dai, Xuanzhi Wang, Kunxue Deng, Xiaoyan Long, Libiao Liu, Xinzhi Zhang, Yongsheng Li, Tao Xu

**Affiliations:** ^1^Department of General Surgery, The First Affiliated Hospital of Anhui Medical University, Hefei, China; ^2^Department of Research & Development, East China Institute of Digital Medical Engineering, Shangrao, China; ^3^Department of Neurosurgery, The First Affiliated Hospital of Anhui Medical University, Hefei, China; ^4^Department of Neurosurgery, The First Affiliated Hospital of USTC, Division of Life Sciences and Medicine, University of Science and Technology of China, Hefei, China; ^5^Department of Mechanical Engineering, Biomanufacturing Center, Tsinghua University, Beijing, China; ^6^Department of Precision Medicine and Healthcare, Tsinghua-Berkeley Shenzhen Institute, Shenzhen, China

**Keywords:** handheld electrospinning, 3D printing, *in-situ* repair, PLA/gelatin, *in-situ*

## Abstract

**Background:** This study aims to design a 3D printed handheld electrospinning device and evaluate its effect on the rapid repair of mouse skin wounds.

**Methods:** The device was developed by Solidworks and printed by Object 350 photosensitive resin printer. The polylactic acid (PLA)/gelatin blend was used as the raw material to fabricate *in-situ* degradable nanofiber scaffolds. Scanning electron microscopy (SEM), Fourier transform infrared spectroscopy (FTIR), X-ray diffraction (XRD), and water vapor permeability test were used to evaluate the material properties of the scaffolds; cytotoxicity test was performed to evaluate material/residual solvent toxicity, and *in situ* tissue repair experiments in Balb/c mouse were performed.

**Results:** The 3D printed handheld electrospinning device successfully fabricates PLA/gelatin nanofibrous membrane with uniformly layered nanofibers and good biocompatibility. Animal experiments showed that the mice in the experimental group had complete skin repair.

**Conclusions:** The 3D printed handheld device can achieve *in situ* repair of full-thickness defects in mouse skin.

## Introduction

The skin is the largest organ of the body, protecting human tissue from external damage. When major trauma, burn/scald, chronic vascular/metabolic diseases, and other skin defects occur, the patient is prone to infection, amputation, systemic complications, and even death due to direct tissue exposure or prolonged healing (Riccio et al., 2019). Prompt and efficient *in-situ* protection and treatment of wounds will provide the necessary environment and regeneration conditions for healing. Dressings play an important role in wound healing. They not only protect the wound from contamination, enhance ventilation, and absorb exudate but also maintain the moisture of the wound, have good tissue biocompatibility and biodegradability, and promote skin regeneration (Feng et al., 2021). In recent years, with the rapid development of biomaterial technology, a large number of excellent biomaterials have shown great potential for clinical application, mainly including synthetic polymer materials [polylactic acid (PLA), polycaprolactone (PCL), polyvinyl alcohol, polyethylene glycol, polyether ether ketone, etc.] and natural polymer materials (gelatin, chitosan, sodium alginate, collagen, hyaluronic acid, silk fibroin, etc.) (Zarei and Soleimaninejad, 2018). Combining the superior characteristics of various materials, a variety of polymer materials, blending, and compounding a variety of polymer material and their bio-manufacturing, cannot only retain the special advantages of the materials but also avoid intrinsic limitations of single materials, so it is widely used in tissue engineering.

Polylactic acid has good biocompatibility, biodegradability (degradation product is non-toxic lactic acid), and mechanical properties. It is a biomaterial approved by the US Food and Drug Administration for *in-vivo* transplantation and thus becomes one of the most commonly used electrospun synthetic polymer materials for soft tissue repair (Paschoalin et al., 2017). However, the simple surface of the PLA molecule is highly hydrophobic and lacks a cell recognition site, which is not conducive to the rapid adhesion, migration, and regeneration of tissue cells. Gelatin is a derivative of collagen that has been partially denatured. It has low immunogenicity, high water retention, and can degrade completely *in-vivo*. It is an ideal substitute of native extracellular matrix (ECM) (Su and Wang, 2015). However, pure gelatin has poor mechanical properties, and the hydrolysis rate is fast; therefore, it is not ideal for *in-vivo* tissue repair. In view of this, PLA and gelatin composites have advantages of both materials, which cannot only maintain the mechanical properties but also delay the degradation rate so that it matches the speed of tissue repair and regeneration. The electrospun nanofibers of polymer materials have good properties, such as porosity, water retention, tissue compatibility, and mechanical properties, and are, therefore, favored in the field of tissue repair, including skin, dura mater, muscle, bone, and cartilage (Su and Wang, 2015; Paschoalin et al., 2017; Zarei and Soleimaninejad, 2018). It has been reported that electrospun nanofibers of PLA/gelatin blends are one of the most ideal material combinations for soft tissue repair applications (Liu et al., 2014; Xu et al., 2015, 2017; Deng et al., 2017). It has been reported in the literature for the repair of brain dura mater, which can promote the migration and colonization of cells and achieve tissue regeneration (Deng et al., 2017).

Traditional electrospinning nanofibers have a relatively complex manufacturing process, requiring high manufacturing conditions, special equipment, a special operation site, and need to be fabricated ahead of time, thus cannot meet the needs of immediate tissue repair in emergency events, such as trauma, burn, and scald. The emergence of portable electrospinning equipment has provided a new solution to this problem. Several researchers have verified that electrospun matrix cannot only safely achieve *in-situ* tissue defect repair but also protect wounds, stop bleeding, and promote tissue regeneration, and have low toxicity (Mouthuy et al., 2015; Xu et al., 2015; Dong et al., 2016; Lv et al., 2016; Liu et al., 2018). Xu reported a miniaturized and integrated battery-powered electronic spinning device. This new device is liberated from the traditional heavy-duty power supply, realizes tight integration of functional components, and can be operated with one hand due to its small size (Xu et al., 2015). Dong developed a personalized handheld electrospinning device and evaluated its characteristics on *in-situ* skin wound repair (Dong et al., 2016). Mouthuy PA and Liu GS fabricated fibrous membranes, using *in-situ* electrospun by a handheld electrospinning apparatus and promised their potential applications in wound healing (Mouthuy et al., 2015; Liu et al., 2018). Zhao reported a portable melt e-spinning gun, which can be *in-situ* electrospun to a wound surface, and prospected this handheld melt e-spinning gun may be used in 3D printing and experimental teaching demonstration aids (Zhao et al., 2020). However, there are still some disadvantages, such as a complex equipment manufacturing process, poor equipment stability, single material with low histocompatibility, and not easy to use. Further improving the stability of the handheld electrospinning device and expanding the applicability of various biomaterials are expected to greatly increase the application of the handheld electrospinning device in the field of instant repair of soft tissues, such as the skin, and promote regeneration.

In this study, we developed a handheld electrospinning device based on 3D printing and used to produce PLA/gelatin electrospun nanofiber scaffolds. Scanning electron microscopy (SEM), Fourier transform infrared spectroscopy (FTIR), X-ray diffraction (XRD), water contact angle, and water vapor transmission rate were measured to assess its biological characteristics, biocompatibility, and toxicity *in-vitro*, and the effect of skin repair on mice with a full-thickness defect, aiming to investigate the feasibility of using nanofiber scaffolds produced by the portable handheld electrospun device. The application potential of instant *in-situ* tissue repair lays the foundation for clinical applications.

## Materials and Methods

### Materials and Reagents

Polylactic acid (molecular weight: 300,000, Cat No. 1604000258) was purchased from Corbion (Netherlands). Gelatin (sourced from pigskin, type A, ~228 g bloom, viscosity (6.67%, 60°C): 3.8 mPas, Cat No. 1010583) was purchased from GELITA (United States, US). Hexafluoroisopropanol (HFIP, Analytical Pure) was purchased from Halocarbon Company (USA). Poly (lactic acid) and gelatin at a mass ratio of 7:3 were added to HFIP, stirred at room temperature for 12 h until completely dissolved, and the spinning solution was prepared to have a total concentration of 8% (w/v). Dulbecco's modified eagle medium (DMEM) high-glucose medium, fetal bovine serum, pancreatin, and phosphate buffer were purchased from Gibco (USA); Alamar Blue was purchased from Maibio (Shanghai, China); the CCK-8 kit was purchased from Dojindo (Japan). The live/dead cell viability staining kit was purchased from KeyGen Biotech (Nanjing, China).

### Cells and Animals

Fetal mouse fibroblasts L929 was purchased from BeiNa Culture Collection (Beijing, China). L929 cells were cultured in DMEM medium, containing 10% fetal bovine serum. The medium was changed every day, and cells were passaged every 3 days. Balb/c mice were purchased from the Nanjing Model Animal Center (Nanjing, China) and fed in an individual ventilated cages (IVC) system. All animal experiments were performed under the supervision of the Ethics Committee of the Tsinghua University and the First Affiliated Hospital of Anhui Medical University. All animal management and operational procedures were strictly performed in accordance with the requirements of the Ethics Committee of the First Affiliated Hospital of Anhui Medical University.

### 3D Printing of Handheld Electrospinning Instrument

3D drawing of the device was developed by Solidworks and exported in the form of standard template library (STL) format, and printed with Object 350 photosensitive resin printer (Stratasys, Israel) for the overall structure. Then electrospinning nozzles, cartridges, high-voltage inverters, power supplies, lines, and switches were installed to make it usable. The handheld electrospinning device is powered by a 12-V rechargeable lithium battery as a voltage generator (Input voltage: 12 V, output voltage: 10 kV, rated power: 5 W, Haorui Electronics, Nantong, China), which can provide up to 10 kV DC high voltage.

We used the pistol design for a more stable grip and more accurate aiming. In order to adapt the solution with different viscosity, we designed the device as palm extrusion. The index finger is fixed on the top, and the thumb drives the palm to squeeze out. Compared with finger extrusion, palm extrusion can achieve greater extrusion force, while less prone to fatigue, suitable for long-term use.

In this design, the commonly used index finger and the thumb are used to ensure the grip, so we designed to use the middle finger to control the high-voltage power switch. The high-voltage power switch is designed to be normally closed so that the electrospinning process can be started and stopped at any time by the force exerted by the middle finger.

A syringe containing the solution is designed at the top of the device for easy loading and replacement. The high-voltage inverter is connected to metal shrapnel through the lead wire, and the metal shrapnel is in close contact with the stainless steel needle at the front of the syringe when the syringe is loaded so that the high-voltage static electricity can be conducted to the solution and generate a high-voltage electrostatic field force.

### PLA/Gelatin Nanofiber Membrane Fabrication

The voltage of the electrospinning device is fixed at 10 kV due to the high-voltage inverter. To create a steady process, the distance between the needle and the skin was set as roughly 10–15 cm; at this range, the nanofibers are generated and form a membrane. The electrospinning time was determined by the size of the defects, 2 cm of the defect just need 10–20 s to cover the whole wound by nanofibers. Because of the rapid process, the thickness of the membrane has a great impact on the electrospinning time and is relatively thin, just about 200–500 μm. The nanofibers were spun and collected after a membrane with appropriate thickness was formed *in-vitro*, and characteristics of nanofiber membrane were performed by SEM, FTIR, and X-ray, according to previous reported methods (Mouthuy et al., 2015; Xu et al., 2015; Dong et al., 2016; Lv et al., 2016; Liu et al., 2018).

### Preparation of Electrospun Membrane Extract

Following the ISO 10993-5 standard, electrospinning membrane (EM) was added to a fresh cell culture medium at a density of 6 cm^2^/ml, immersed at 37°C for 24 h, and then the extract was collected. The extract was made into a working solution by diluting it with the fresh cell culture medium at a 100, 50, and 25% volumetric ratio. The cell culture medium was used as a negative control (NC), and 5% DMSO was used as a positive control (PC).

### CCK-8 Cytotoxicity Assay

L929 cells in a logarithmic growth phase were made into cell suspension, and 100-μl cell suspension with 4 × 10^4^ cells/ml was seeded into individual wells of a 96-well plate, and incubated overnight at 37°C with 5% CO_2_; then, the medium was aspirated from each well, and 100 μl of the extract to be tested was added and cultured for 24 h; 100 μl of CCK-8 reaction solution (90 μl of medium + 10 μl of CCK-8) was added to each well. For the PC and NC groups, after the medium was aspired off, instead of the extract, 5% DMSO and a cell culture medium was added, with all other steps kept the same. Empty wells were added with the reaction solution as a blank control and incubated at 37°C for 2 h. The optical density (OD) value at a 450-nm wavelength of each well was measured with a microplate reader. The formula calculates the cell relative survival rate (RGR) = [mean (OD of experimental group – OD value of a blank group)/mean (OD value of control group – OD value of a blank group)] %.

### Mechanical Properties

The samples were cut into a rectangle with a width of 10 mm and a length of 50 mm; both ends of the sample were fixed with clamps with a separation distance no <10 mm for tensile strength testing. The ultimate tensile force and elongation at the break were measured, using a uniaxial tensile force at a rate of 50 mm/min. The ultimate tensile strength was obtained by dividing the ultimate tensile force by the cross-sectional area of the samples.

### Water Vapor Transmission Rate

According to the EU EN 13726-2 (2002) standard, Part 2 of the main wound dressing test method: PLA nanofiber membrane, PLA/gelatin nanofiber membranes, and gelatin nanofiber membrane were tested for the water vapor transmission rate.

### Contact Angle

The water contact angle of the electrospun membrane was measured, using a German OCA-20 video optical contact angle meter. About 0.2 ml of deionized water was added to the EM, and, after the droplet was stabilized, a photograph was taken, and the contact angle was measured. Each group was repeated five times and averaged.

### Alamar Blue Assay

The electrospun membrane was cut into pieces to match the size of the 24-well plate with a punch and sterilized by irradiation with 60 Gy Co60 gamma ray and placed in a 24-well plate with 2 × 10^5^ cells/ml of L929 cell suspension per well. About 1-ml culture was added, and cells were cultured overnight. The Alamar blue stock solution was diluted with phosphate buffer saline (Alamar blue: PBS = 1:9) to form working solution. About 1 ml of working solution was added to each well, incubated for 1 h at 37°C; and 100 μl of the working solution was added to individual wells of a 96-well plate. The absorbance (OD value) at 570 and 630 nm was read by the Microplate Reader (Biotek, ELX800); then, a cell-containing scaffold was washed two times with PBS, and a fresh culture medium was added to continue the culture.

### Live/Dead Staining

L929 cells grown in 24 well plates were stained with live/dead staining on the third day of the culture. About 10-ml PBS was used to dilute the live/dead staining solution to obtain 2-μM calcein AM and 8-μM PI working solution; 1 ml of which was added to individual wells, incubated for 15 min at room temperature, washed in PBS, and observed under a fluorescence microscope. For each sample, five fields of view were randomly selected and viewed at 200×. Images were taken for analysis. For each group, this measurement was determined for three samples. The number of green (live) and red (dead) cells in each image was recorded to calculate cell viability (live cell/total of live and dead cells × 100%), and the average for each group was obtained. SEM: The cells were seeded on an electron-spun scaffold for 72 h and then fixed in 3% glutaraldehyde solution, precooled at 4°C for 12 h, washed three times in PBS, and then dehydrated. The specific steps were: a 1:1 volumetric ratio of acetone/isoamyl acetate for 10 min, 100% isoamyl acetate for 30 min; 50% (v/v) aqueous acetonitrile, followed by 70, 80, 90, 95, and 100% acetonitrile in that order. The membrane was immersed for 20 min in each solution, and, finally, vacuum dried. The dried sample was fixed on the sample holder by a conductive adhesive, and a platinum layer was sprayed on the surface of the stent. The samples were observed and photographed at a voltage of 5 kV.

### Animal Experiments

All animal experiments were conducted according to the guidelines of the National Institutes of Health on the use of laboratory animals and were carried out under the supervision of the Animal Research Ethics Committee of the First Affiliated Hospital of Anhui Medical University. A total of 18 Balb/c mice (25–30 g) were randomly divided into groups for the experiment, nine mice in the experimental group for *in-situ* electrospinning, and nine other mice as blank control. Mice were anesthetized with pentasorbital sodium (40 mg/kg), and the back hair was shaved, and then a full-thickness skin defect, having a diameter of 2.0 cm, was surgically produced. Mice in the experimental group were subjected to *in-situ* electrospinning and following by gauze dressing, in which the spray range (distance and size) was manually adjusted so that the nanofibers uniformly sprayed over the wound surface, and the control group only used conventional gauze dressing. All the mice were resuscitated and continued regular feeding. The incision was observed weekly until the wound was no longer visible.

### Pathological Feature

The experimental mice were sacrificed after 8 weeks. Full-thickness skin of the surgical area was fixed with 4% paraformaldehyde, embedded in paraffin after dehydration, and sliced to 5-μm sections. Dewaxing was performed, following the protocol; hematoxylin-eosin (HE) staining and masson trichrome staining were performed, and the sections were then observed with light microscopy.

### Statistical Analysis

The data were analyzed and plotted, using the GraphPad prism 8.0.1 software. The data were expressed as mean ± standard deviation (*mean* ± *SD*). *T*-tests or one-way ANOVA was used to analyze statistical significance. The difference was statistically significant at ^*^p < 0.05, ^**^p < 0.01, and ^***^p < 0.001.

## Results

### A 3D Printed Handheld Electrospinning Device

**Figure 1:**
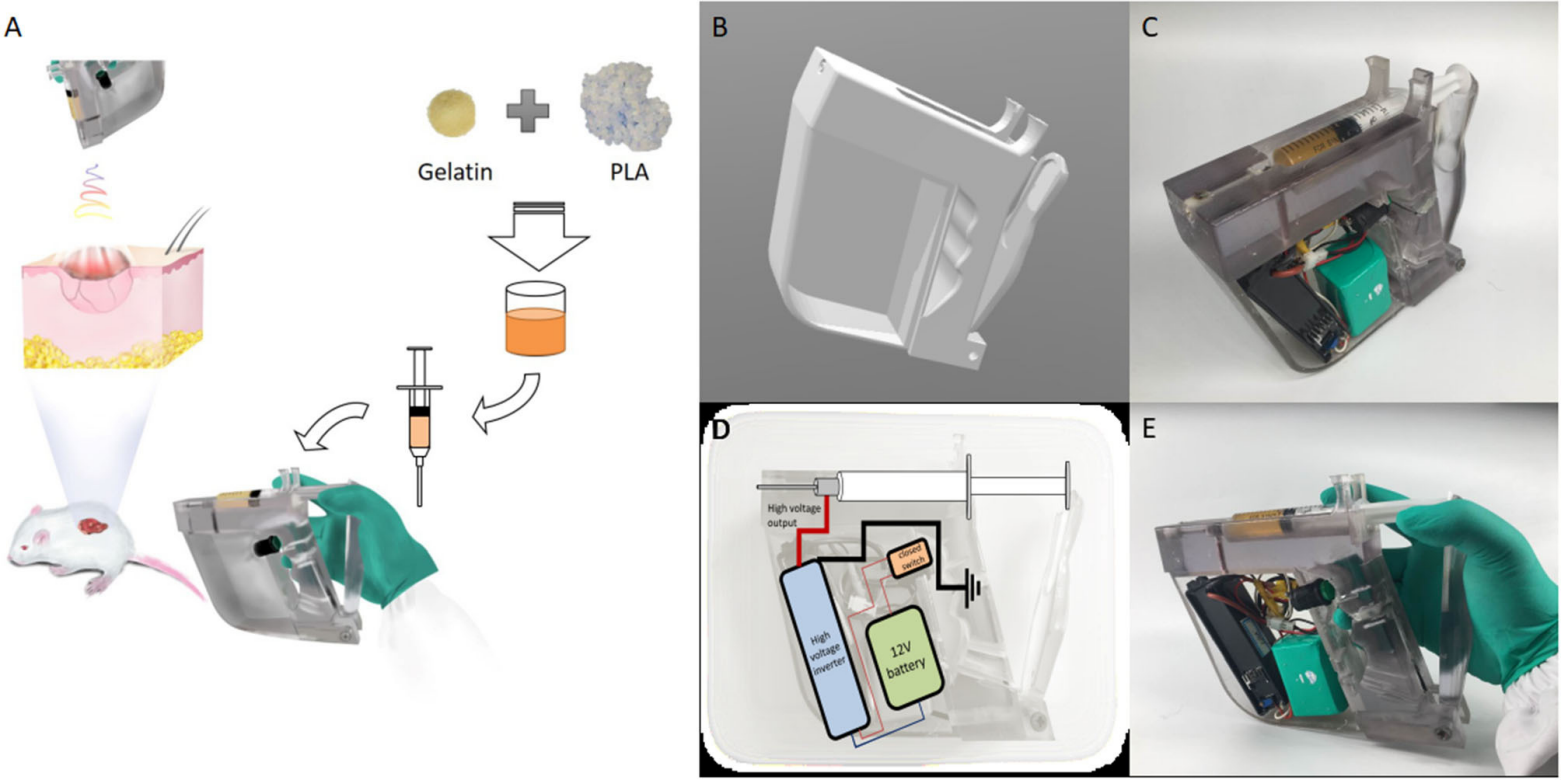
The handheld electrospinning device. **(A)** A schematic diagram of the working state; **(B)** CAD design of the 3D printed device; **(C)** The device after installation; **(D)** Circuit design of the handheld electrospinning device; **(E)** Working posture of the handheld electrospinning device.

In this study, a handheld electrospinning device was designed. The schematic diagram of the experimental process is shown in Figure 1A. The design is a gun-type and ergonomic device (Figure 1B), which can be held and operated by one hand. This device was printed by 3D printer (Stratasys, Israel), using the photosensitive resin material. The 3D printed handle was then equipped with a power supply, a high voltage inverter, a control switch, a power supply line, an injector, and a syringe needle, which was connected to the outlet of the high-voltage inverter, and the device was ready to use, as shown in Figure 1C. The circuit design of the handheld electrospinning device is shown in Figure 1D. At the same time, taking the actual use as a starting point, the extrusion part of the handheld electrospinning is designed as manual extrusion, in which the control switch and the extrusion part are separate. To prevent the needle from plugging, first, extrusion should be performed until the spinning solution fills to the tip of the needle; then, the high-voltage inverter switch can be turned to start the electrospinning process. The way of holding the device during use is shown in Figure 1E.

### Properties of Electrospun Nanofiber Membranes Prepared by the Handheld Device

#### Scanning Electron Microscopy

**Figure 2:**
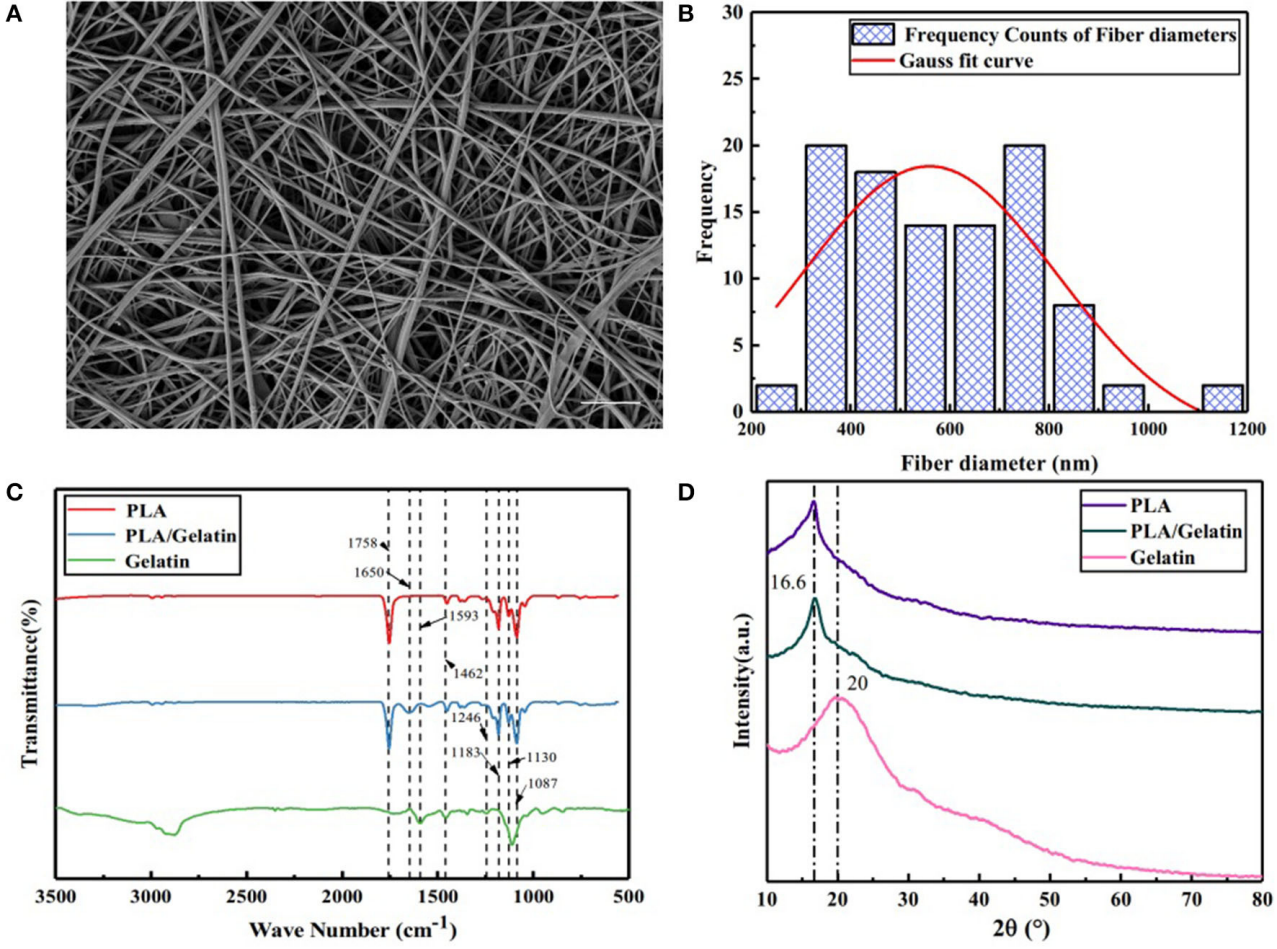
Characteristics of electrospun PLA/gelatin materials. **(A)** Representative SEM image, showing the microstructure; **(B)** nanofiber diameter distribution; **(C)** FTIR-ATR spectrum; **(D)** XRD spectrum. Scale bar A 10μm.

Scanning Electron Microscopy images of an electrospun scaffold prepared *in-situ* by the handheld device are shown in Figure 2A. It can be seen that, after *in-situ* electrospinning, the PLA/gelatin spinning solution was stretched and cured into relatively uniform nanofibers with an average fiber diameter of 584.44 ± 188.43 nm. Statistical analysis showed that the diameter distribution conformed to Gaussian normal distribution, as shown in Figure 2B.

#### Fourier Transform Infrared Spectroscopy

Fourier transform infrared spectroscopy analysis results of PLA/gelatin nanofibers, PLA nanofibers, and gelatin nanofibers are shown in Figure 2C. For PLA nanofibers, the absorption peaks appearing at 1,758 and 1,452, 1,183, 1,130, and 1,087 cm^−1^ corresponded to the stretching vibration absorption peak of C=O, the stretching vibration absorption peak of –CH3, the C–O anti-symmetrical stretching vibration absorption peak, the C–O–C stretching vibration absorption peak, and the C–O symmetrical stretching vibration absorption peak, respectively. Absorption peaks appearing at 1,593, 1,462, and 1,246 cm^−1^ of gelatin nanofibers corresponded to the N–H deformation vibration absorption peak of amide I band, the N–H stretching vibration absorption peak of amide II band, and the N–H stretching vibration absorption peak of the amide III band, respectively. When the two materials were combined, the PLA/gelatin nanofibers basically followed the infrared characteristics of the PLA nanofibers, and the characteristic absorption peaks attributable to gelatin disappeared. It was indicated that the addition of a small amount of gelatin did not have a significant effect on the characteristic groups of the PLA; it was found that PLA/gelatin nanofibers exhibited an absorption peak at 1,650 cm^−1^, corresponding to the absorption peak of the C = O stretching vibration of the amide I band, indicating that a weak chemical effect was generated between gelatin and PLA, which was helpful in even distribution of gelatin in PLA. The attribution of peaks was shown in Table 1.

**Table 1:** The attribution of peaks.

**Materials**	**Wave number (cm** ^**−1**^ **)**	**Groups**	**Molecular vibration type**
PLA	1,758	C=O	Stretching vibration
	1,452	–CH3	Stretching vibration
	1,183	C–O	Anti-symmetrical stretching vibration
	1,130	C–O–C	Stretching vibration
	1,087	C–O	Symmetrical stretching vibration
Gelatin	1,593	N–H of amide I band	Deformation vibration
	1,462	N–H of amide II band	Stretching vibration absorption
	1,246	N–H of amide III band	Stretching vibration
PLA/Gelatin	1,650	C=O of amide I band	Stretching vibration

#### X-Ray Diffraction

The XRD patterns of PLA/gelatin nanofibers, PLA nanofibers, and gelatin nanofibers are shown in Figure 2D. It can be seen that PLA nanofibers exhibited a crystallization peak at the 16.6° position, corresponding to the (200) crystal plane of PLA. Gelatin nanofibers appeared as taro peaks around 20°. When the two were combined, the PLA/gelatin nanofibers only exhibited a crystallization peak at the 16.6° position, and there was no significant difference in the XRD curve when compared with that of PLA nanofibers, indicating that the addition of gelatin had no effect on the crystallization performance of the composites. Quantitative results from XRD were performed, smooth processing in the origin software, and the processing method was FFT Filter.

#### Stress/Strain Curves of the Gelatin, PLA, and PLA/Gelatin

**Figure 3:**
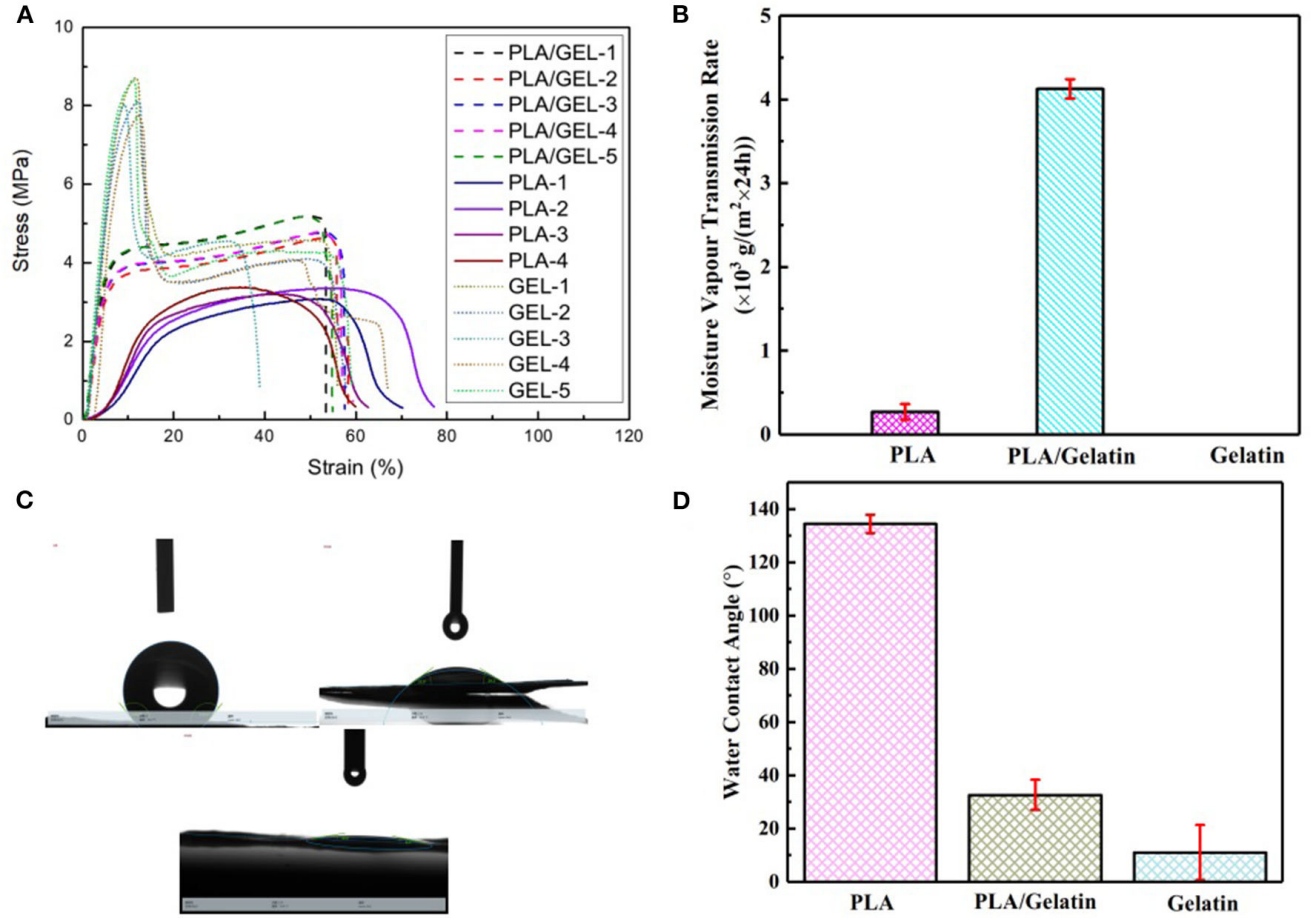
Properties of electrospun PLA/gelatin materials. **(A)** Stress/strain relation curves of the gelatin (red), PLA (green), and PLA/gelatin (blue); **(B)** water vapor transmission rate; **(C,D)** water contact angle.

Results showed that (1) Gelatin had a lower tensile strength and lower elongated deformation than the PLA and PLA/gelatin; (2) PLA had lower elongated deformation than the PLA/gelatin; (3) PLA/gelatin had lower tensile strength but softer than PLA, which suggested appropriate properties on tensile strength and elongated deformation, as shown in Figure 3A and Table 2.

**Table 2:** Stress/strain properties.

**Sample**	**Tensile strength**	**Elongated deformation**	**Young's modulus**
	**at break**	**at break**	
	**(MPa)**	**(%)**	**(MPa)**
PLA	3.25 ± 0.14	67.53 ± 7.78	0.20 ± 0.05
PLA/Gelatin	4.91 ± 0.26	56.71 ± 2.48	1.03 ± 0.07
Gelatin	8.24 ± 0.41	55.79 ± 10.34	1.37 ± 0.12

#### Water Vapor Transmission Rate

The water vapor transmission rate of PLA/gelatin nanofibers, PLA nanofibers, and gelatin nanofibers is shown in Figure 3B. As a dressing on the skin, in addition to the need to protect the wound surface from the external environment, the *in-situ* electrospun nanofibers also need to have certain permeability. It has been found through testing that PLA nanofibers have a hygroscopicity of only 0.27 ± 0.09 × 10^3^ g/(m^2^ × 24 h) due to their hydrophobicity. Since gelatin nanofibers are soluble in water, when they are subjected to the moisture permeability test, the membranes were rapidly wetted by water and then ruptured, and the moisture permeability could not be obtained. Therefore, the actual application value of *in-situ* electrospinning of gelatin nanofibers is not available. When the two were combined, the moisture permeability of PLA/gelatin nanofibers can reach 4.21 ± 0.11 × 10^3^ g/(m^2^ × 24 h). In summary, PLA/gelatin nanofiber material is suitable for skin repair and can be electrospun *in-situ*, so PLA/gelatin nanofibers were selected for subsequent biological tests.

#### Water Contact Angle

The statistics of water contact angle of PLA/gelatin nanofibers, PLA nanofibers, and gelatin nanofibers are shown in Figures 3C,D. The water contact angle of PLA nanofibers is 134.48 ± 3.43°, which belongs to the range of hydrophobic materials. The gelatin nanofibers used in this study were not cross-linked, so water droplets quickly dispersed when they were exposed to gelatin nanofiber membranes; furthermore, gelatin nanofibers were easily soluble in water, and, consequently, water contact angles on gelatin samples could not be measured. By increasing the thickness of the nanofiber membrane and shortening the photograph time, the water contact angle of the gelatin nanofiber was 11.02 ± 10.34°, belonging to the range of hydrophilic materials. When the two were combined, the water contact angle of the PLA/gelatin nanofibers was measured to be 32.68 ± 5.68°, belonging to the range of hydrophilic material, and this water contact angle was within the range of 30–50°. Therefore, the water contact angle of PLA/gelatin nanofibers satisfies the requirement for cell adhesion (van Wachem et al., 1987).

### Biological Characteristics of Handheld Electrospun Nanofiber Membranes *in-vitro*

#### Cytotoxicity

**Figure 4:**
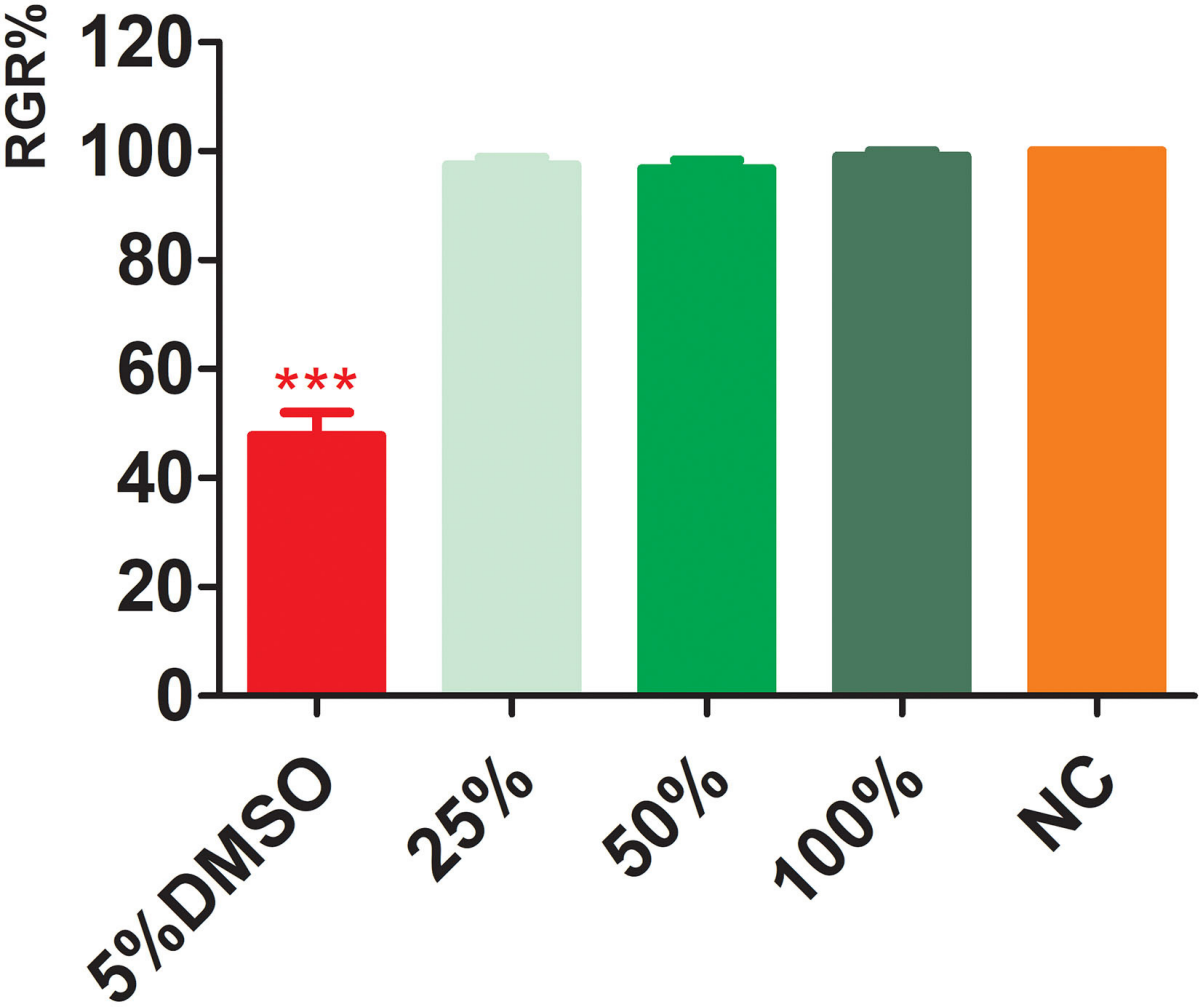
Cytotoxicity of electrospun membrane extracts. DMSO, dimethyl sulfoxide; NC, negative control. ^***^p < 0.001.

The results of CCK-8 cytotoxicity experiments with electrospun membrane extracts (Figure 4) showed that the relative proliferation fractions of cells cultured in 100, 50, and 25% concentrations were 97.42 ± 1.40, 96.73 ± 1.60, and 99.09 ± 0.94%, respectively. Compared with the NC, there was no significant difference (*p* > 0.05), indicating, there was no significant cell cytotoxicity of the extracts. The relative proliferation rate of cells in the PC DMSO group was 47.79 ± 4.30%; the difference in cytotoxicity was statistically significant with a PC group (*P* < 0.001).

#### Cell Survival: L929 Cells Were Seeded on the Electrospinning Membrane

**Figure 5:**
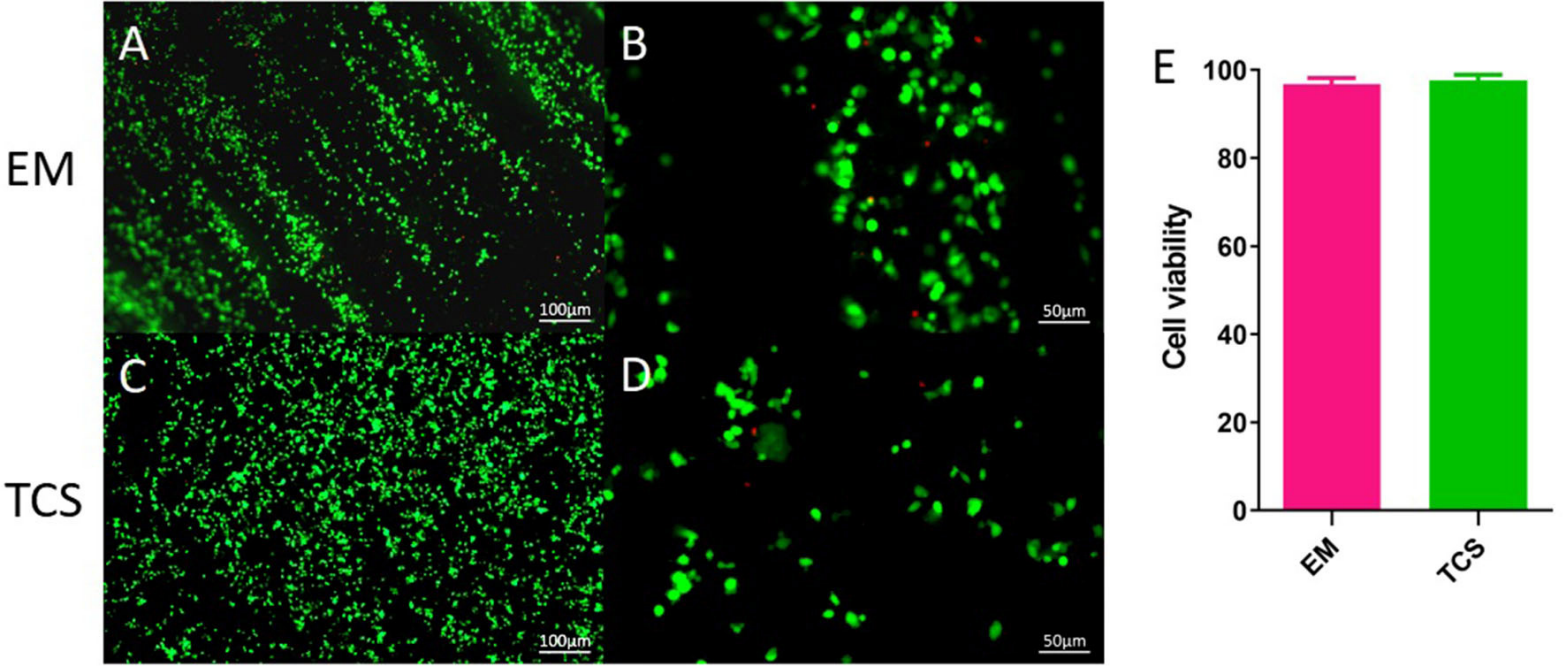
Cell survival. **(A–D)** Live/dead staining; **(E)** cell viability. Scale bar: **(A,C)** 100μm, **(B,D)** 50μm.

Live/dead staining was performed on day 3 (Figures 5A–D). Compared with traditional culture slides (TCS, cells cultured on glass slides), the cell viability in the EM and traditional culture slides was 96.78 ± 1.41 and 97.59 ± 1.32%, respectively (Figure 5E). There was no significant difference between the two groups (*P* > 0.05).

#### Cell Morphology

**Figure 6:**
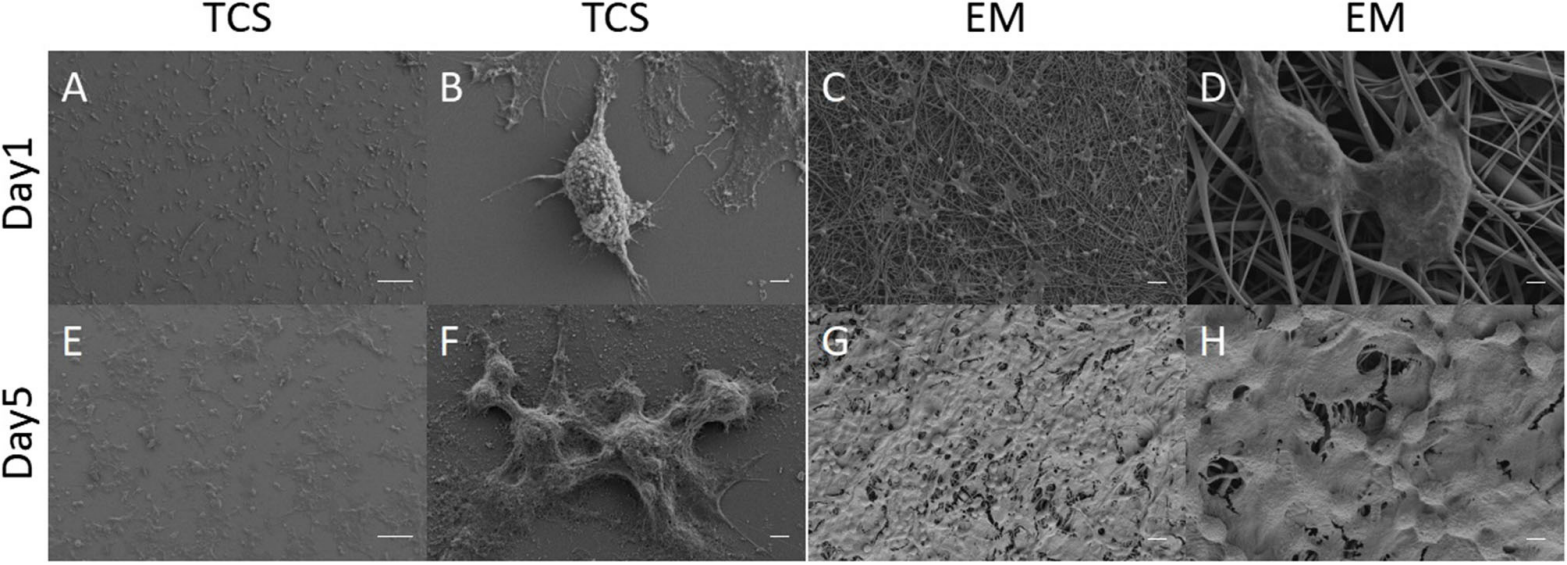
SEM: Cell morphology of L929 grown on EM **(C,D,G,H)** and TCS **(A,B,E,F)** on day 1 and day 5. Scale bar: **(A,B)** 100μm, **(B,D,F,H)** 2μm, **(C,G)** 20μm.

L929 cells were seeded and observed under electron microscopy (Figures 6A–H). In the control of TCS (Figures 6A,B,E,F), the cells protruded from pseudopodia adhered to culture slides with good morphology. The cells on the EM partially adhered to the surface of the EM and penetrated into the scaffold, and the pseudopodia spread well along the nanofibers (Figures 6C,D,G,H).

#### Cell Proliferation

**Figure 7:**
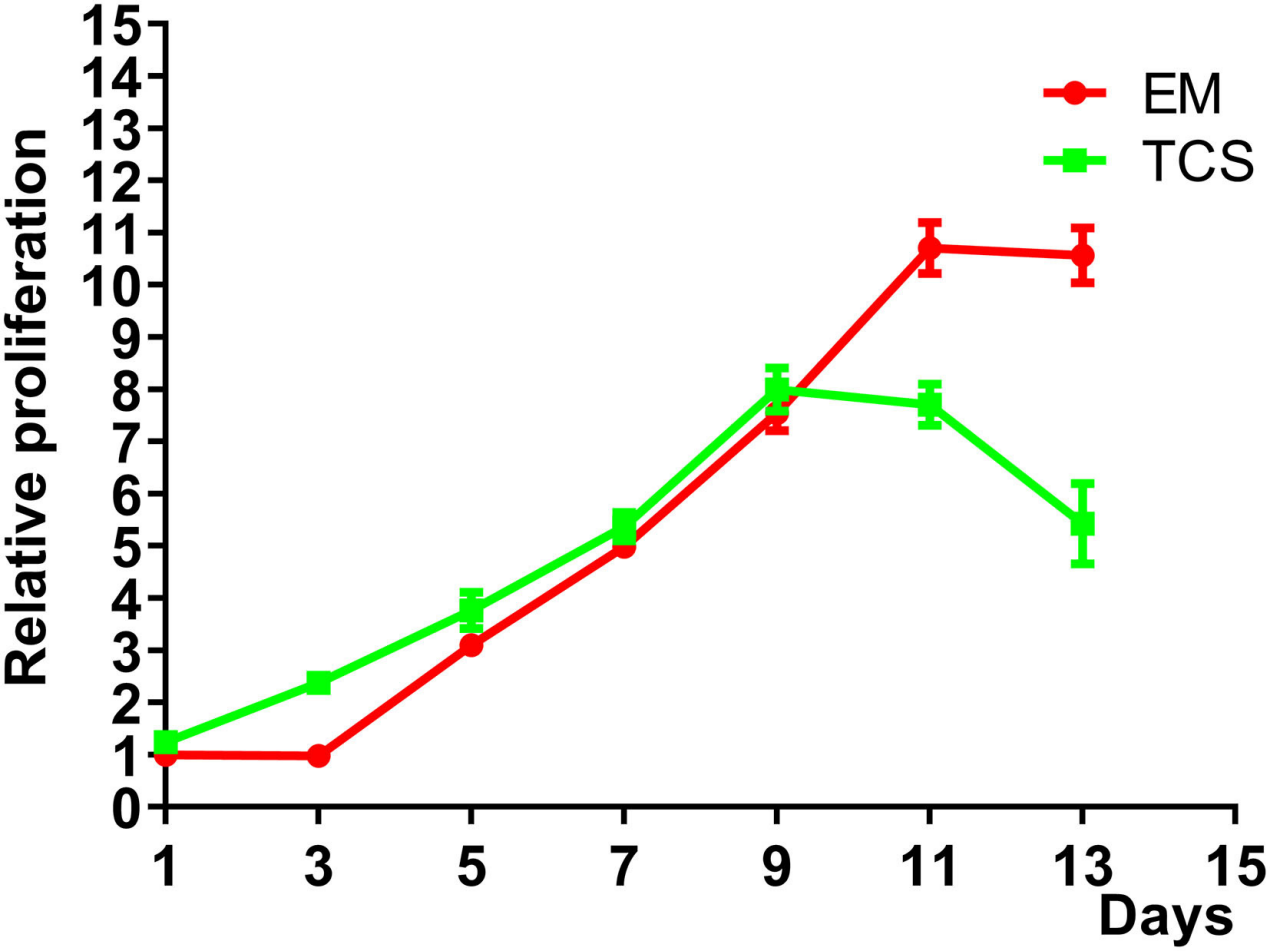
Cell proliferation curves of L929 cells on EM, compared with that of culturing on TCS.

The 3D proliferation results of mouse embryonic fibroblasts L929 in EM were compared with that of TCS (Figure 7). The initial cell proliferation rate was relatively slow, but there was no significant difference in the doubling time; the cell proliferation rate of both groups reached a plateau during the culturing period, with the EM reached it earlier. The proliferative activity of the TCS group decreased on the ninth day and showed obvious apoptosis. The EM group still maintained proliferative activity up to 13 days of culturing. This experiment shows that the cells in the EM group maintained their activity comparable to that of the TCS group, and a relatively large amount of proliferation surface could maintain a longer cell proliferation activity.

### *In-situ* Repairing With Electrospun Nanofibers Prepared by the Handheld Device

#### Animal Experiments

**Figure 8:**
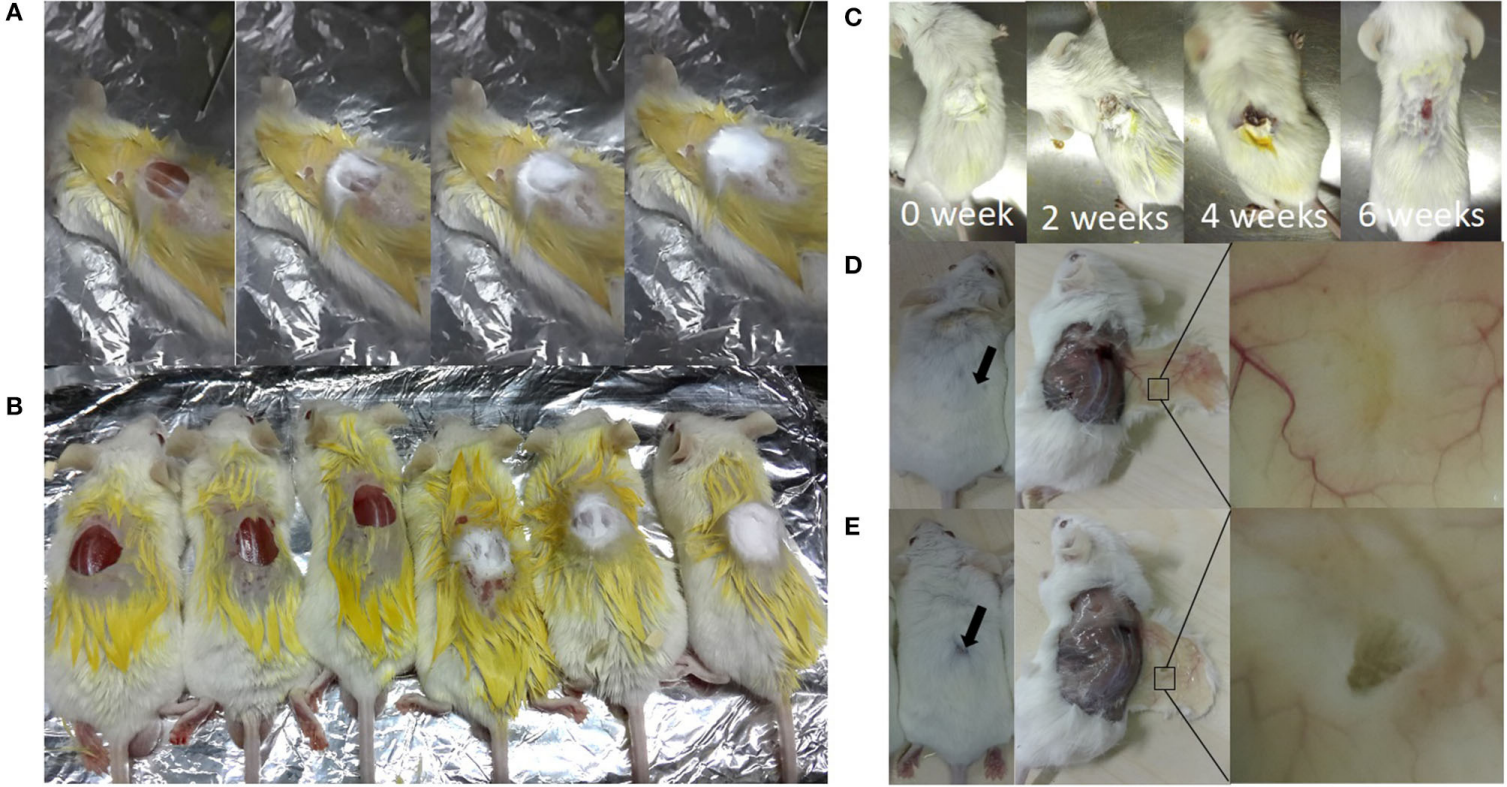
*In-situ* repair of skin defects in Balb/c mouse. **(A)** A process of *in-situ* repair of skin defects; electrospinning was performed after wound creation immediately; **(B)** Balb/c mouse after repairing (right, three mice) or not (the NC group; left, three mice); **(C)** dynamic observation of wound healing; **(D)** 8 weeks after electrospinning *in-situ* repair, showing skin defects were completely repaired; **(E)** control group, showing incomplete repair after surgery for 8 weeks.

*In-situ* repairing with electrospun nanofibers by the handheld device was performed, as shown in Figure 8A, and the repair process of skin defects, using handheld electrospun nanofibers, is shown in Figure 8B. The repair process of the skin defects was observed dynamically and the EM falloff in about 6 weeks (Figure 8C). After 8 weeks, the mouse skin defect repaired with nanofiber membrane healed completely, and their hairs regenerated and completely covered the defects (Figure 8D); in the control group, the skin defects were visible to the naked eye, and obvious defects were found after skin opening. It was considered as not healed (Figure 8E).

#### Pathological Features

**Figure 9:**
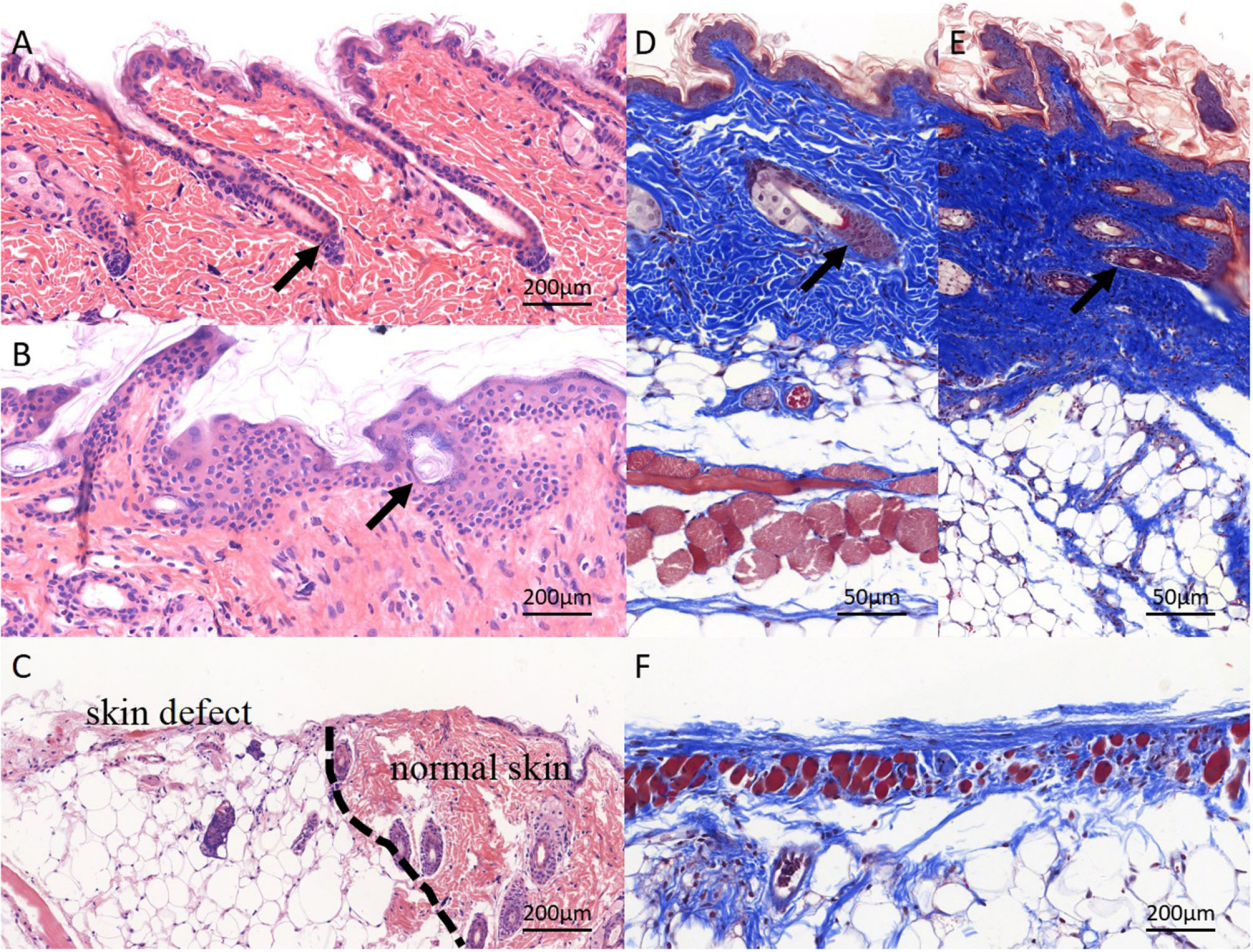
Pathological staining of repaired skin defects in Balb/c mouse. **(A,D)** HE and masson staining of normal skin, and the skin structure is complete, and the layers are clear; **(B,E)** electrospun nanofiber membranes repaired skin. The structure of skin tissue is disorder, several hair-follicle-like structures can be seen (black arrow), layers are clear basically, the newly deposited collagen structure is dense, and the dermal structure is completely covered; the **(C,F)** control group. No obvious skin structure was observed, collagen production was weak, and skin defect was not covered by dermis.

Pathological staining of the normal skin was shown in Figure 9A; the skin and its ancillary organs remained intact; the repaired skin from experimental mice remained intact and showed full-thickness repair; the boundaries between layers were clearly identified; and some hair follicles were visible, with hair regrowth at the edges of defects (Figure 9B). In the control group, the edge of the skin defect was seen, and the border of the defect was dominantly fat cavities without skin (Figure 9C). The pathological sections of all the animals showed no obvious inflammatory reaction and infiltration of immune cells. Masson trichrome staining showed that the normal skin and collagen fibers were intact (Figure 9D). In the electrospun nanofiber membrane repaired group, the mouse skin was relatively intact, the skin was completely covered, and the proliferated collagen fibers were thicker than that of normal skin (Figure 9E). Collagen fibers were also seen in the control group, but without skin covering, and the repair effect was significantly worse than that of the experimental group (Figure 9F). It can be seen that electrospun nanofiber membranes prepared by the handheld device helped to achieve full-scale repair of mouse skin.

## Discussion

The development of handheld portable electrospinning device has been reported to fabricate nanofibrous dressing for wound care (Liu et al., 2018). Here, we produce a 3D printed handheld electrospinning device, which has the characteristics of fast production, portable, stable and economical. The manufactured nanofiber membrane has good material properties, including uniform distribution of a nanofiber diameter, good mechanical properties, hydrophilic properties, degradable properties, and good biocompatibility, and there is no significant difference from the nanofiber membrane obtained with classical electrospinning devices, which can meet the requirements of skin external dressings (Kailani et al., 2016). The nanofiber membrane has demonstrated superior wound repair effects, especially in the rapid coverage of skin wounds, reducing exudation and promoting wound repair and healing, etc., showing great potential for clinical application. Fourier transform infrared spectroscopy analysis results of PLA/gelatin nanofibers were indicated that the addition of a small amount of gelatin did not have a significant effect on the characteristic groups of the PLA. One of the main reasons for the difficulty of skin defect regeneration is the lack of migration and adhesion of the dermal cells. The cells cannot cover the wound through crawling and tissue regeneration, making it difficult to close. Polymeric nanofiber scaffolds are widely used in skin tissue regeneration (Sun et al., 2018). To enhance skin regeneration, a variety of methods, including using chemical-induced methods, such as growth factors, chemokines, microRNAs, and using nanofiber materials with a physically biomimetic structure (Lien et al., 2009), are used to stimulate skin regeneration and functionalization. Gelatin-based material has good physical properties; it is not only rich in cell attachment sites but also can mimic the characteristics of the ECM in the tissue microenvironment, promote cell–cell and cell–matrix interactions, and provide an ideal matrix microenvironment for skin cell growth (Kim et al., 2008). A PLA/gelatin blend is a good candidate for electrospinning, as PLA provides the strength and elasticity of fiber scaffolds, and gelatin provides better tissue compatibility and promotes cell spreading and coverage.

The PLA/gelatin nanofiber scaffold made by handheld electrospinning device mimicked the microscopic structure of the natural ECM and had a good promoting effect on cell adhesion, proliferation, and migration. It was deposited *in-situ* at the skin defect. It not only protected the wound from external contamination in time but also kept the wound from becoming wet and provided an environment conducive to skin regeneration. It also provided attachment points for dermal cell migration and spreading, as well as support for angiogenesis and skin regeneration (Wang et al., 2009; Chiou et al., 2013; Yang et al., 2020). PLA/gelatin nanofiber scaffolds have even been commercialized in soft tissue repair applications for meningeal repair (Liu et al., 2018), which is sufficient to demonstrate the safety of PLA/gelatin in human and animal tissue repair applications. For the fibrous matrix composed of PLLA and GE, Xiaoping Yang (Yang et al., 2011) indicated that composition of PLLA and GE exhibited a continuous structure on account of the phase separation occurring in fiber solidification. Hydrophobic interactions were identified between PLLA and GE, which led to the remarkably increasing hydrophilicity and lowered GE solubility. Furthermore, most of the cross-linkers have poor biocompatibility, which could lead to second damage to the wound. For those reasons, the GE of this research has not been cross-linked.

Handheld electrospinning devices have integrated, portable, and fast-application features. Long et al. (Mouthuy et al., 2015; Xu et al., 2015; Dong et al., 2016; Lv et al., 2016) reported using a self-manufactured handheld electrospinning device for *in-situ* repair of meninges, liver, and skin, which significantly promoted tissue healing, but there are still limitations. (1) The device was designed for one-hand gripping. However, single-finger pressings are hard for researchers with relatively small palms and insufficient finger strength to use; (2) The material used was, basically, the highly hydrophobic PCL, which provided a microenvironment for maintaining water retention and for tissue regeneration. However, it was not ideal for cells. In contrary, the equipment used in our study was fabricated based on CAD drawings. The shape and housing device manufactured, using 3D printing, conformed to the ergonomic design, and it was convenient for one-handed handheld operation. 3D printing new medical devices can be used in several departments, such as anesthesia, orthopedics, dental, and neurosurgery (Cahuana-Bartra et al., 2020; Faldini et al., 2020; Lan et al., 2020).

In this study, we demonstrated the superiority of electrospun PLA/gelatin nanofiber membranes prepared by the handheld device in skin repairing. Results from a variety of tests, including SEM, FTIR, XRD, hydrophilicity, and breathability confirmed the superior material properties of PLA/gelatin nanofibers, which meet the requirements of *in-situ* electrospinning and confirmed it was an ideal skin repair material. Taking into account that the *in-situ* repair with electrospun nanofibers may have solvent residues affecting cell viability, CCK-8 assay was used to assess cytotoxicity, and the results showed no significant difference between the experimental and control groups. Experiments of *in-vitro* culturing of fetal rat fibroblasts on nanofiber membranes showed that the cells can maintain the activity equivalent to petri dish-cultured cells on the membrane, had a faster proliferation rate, and maintained a longer proliferation time. Gross morphology, pathological HE, and masson trichrome staining results showed that the electrospun nanofiber membrane repaired group had full wound healing and hair follicle regeneration. The skin of mice in the control group did not achieve full-thickness healing, fully confirming the potential of using electrospun nanofiber membranes prepared by the handheld device for skin repair.

## Conclusion

Through the self-designed and 3D-printed handheld electrospinning device, the PLA/gelatin nanofiber membrane can be instantly prepared for *in-situ* repair of skin defects; *in-vitro* and *in-vivo* and experiments have demonstrated that PLA/gelatin has superior material properties and biocompatibility, which shows its potential application for skin defect repair.

## Data Availability Statement

The original contributions presented in the study are included in the article/Supplementary Material, further inquiries can be directed to the corresponding author/s.

## Ethics Statement

The animal study was reviewed and approved by the Ethics Committee of the First Affiliated Hospital of Anhui Medical University.

## Author Contributions

HC contributed to study design, the animal experiment, and writing of the paper. HZ contributed to 3D printing of the handheld electrospinning device. YS and XD contributed to data analysis and graph drawing. XW contributed to the cell experiment. LL, KD, and XL contributed to data collection and 3D modeling software handling. XZ contributed to the first and the final drafts of the paper. YL and TX contributed to study design and revision of the final draft of the paper. All authors contributed to the article and approved the submitted version.

## Conflict of Interest

The authors declare that the research was conducted in the absence of any commercial or financial relationships that could be construed as a potential conflict of interest.

## Publisher's Note

All claims expressed in this article are solely those of the authors and do not necessarily represent those of their affiliated organizations, or those of the publisher, the editors and the reviewers. Any product that may be evaluated in this article, or claim that may be made by its manufacturer, is not guaranteed or endorsed by the publisher.

## Abbreviations

PLA: polylactic acid
SEM: scanning electron microscopy
FTIR: Fourier transform infrared spectroscopy
XRD: X-ray diffraction
DMEM: Dulbecco's modified eagle medium
PBS: phosphate buffer saline
HE: hematoxylin-eosin.
