# Supplementary figures and images for: Instant in-situ Tissue Repair by Biodegradable PLA/Gelatin Nanofibrous Membrane Using a 3D Printed Handheld Electrospinning Device
Source: Front Bioeng Biotechnol. 2021 Jul 28;9:684105. doi: 10.3389/fbioe.2021.684105 (PMC8355707; doi:10.3389/fbioe.2021.684105)

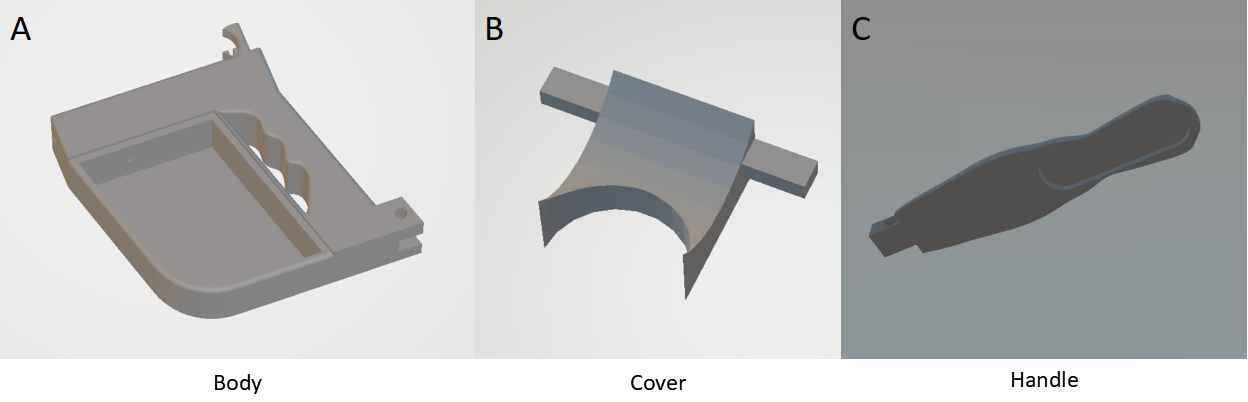

Supplement: Supplementary file 2 [file Image_1.TIF]

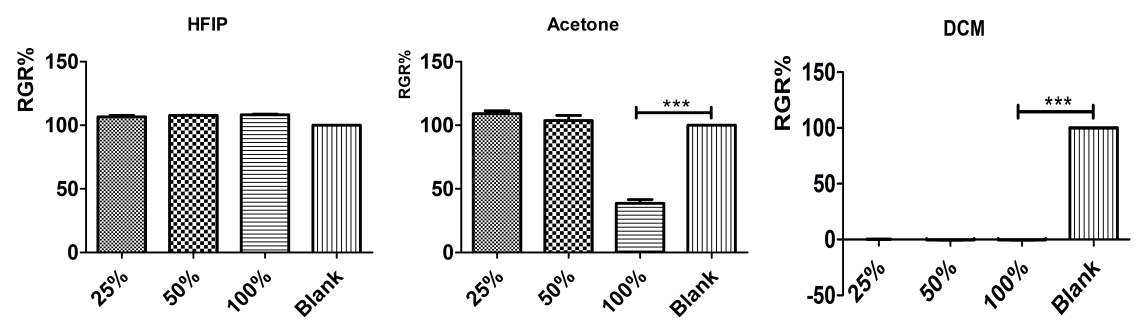

Supplement: Supplementary file 3 [file Image_2.TIF]

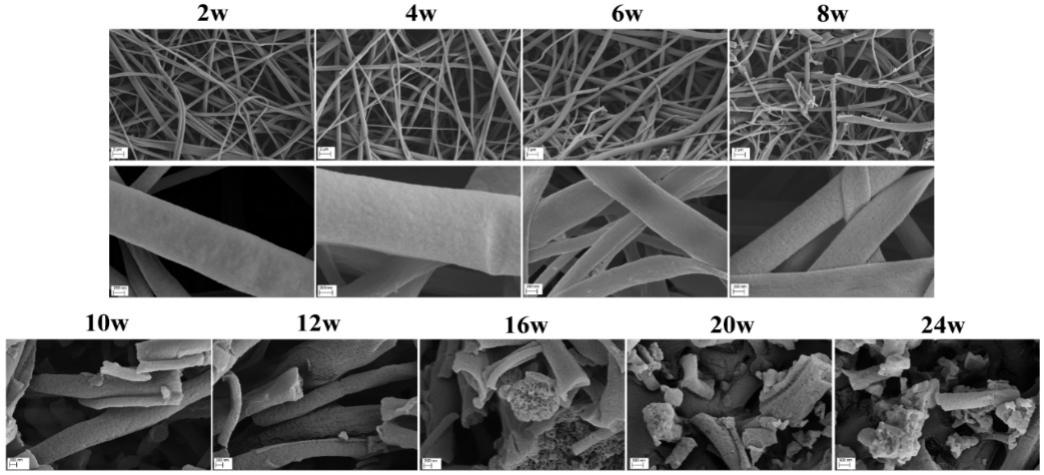

Supplement: Supplementary file 4 [file Image_3.TIF]
